# Supplementary material for: Insight into Glyproline Peptides’ Activity through the Modulation of the Inflammatory and Neurosignaling Genetic Response Following Cerebral Ischemia–Reperfusion
Source: Genes (Basel). 2022 Dec 16;13(12):2380. doi: 10.3390/genes13122380 (PMC9777888; doi:10.3390/genes13122380)
Supplement: Supplementary file 1 [file genes-13-02380-s001.zip › Supplementary Figure S1.pptx]

## Slide 1
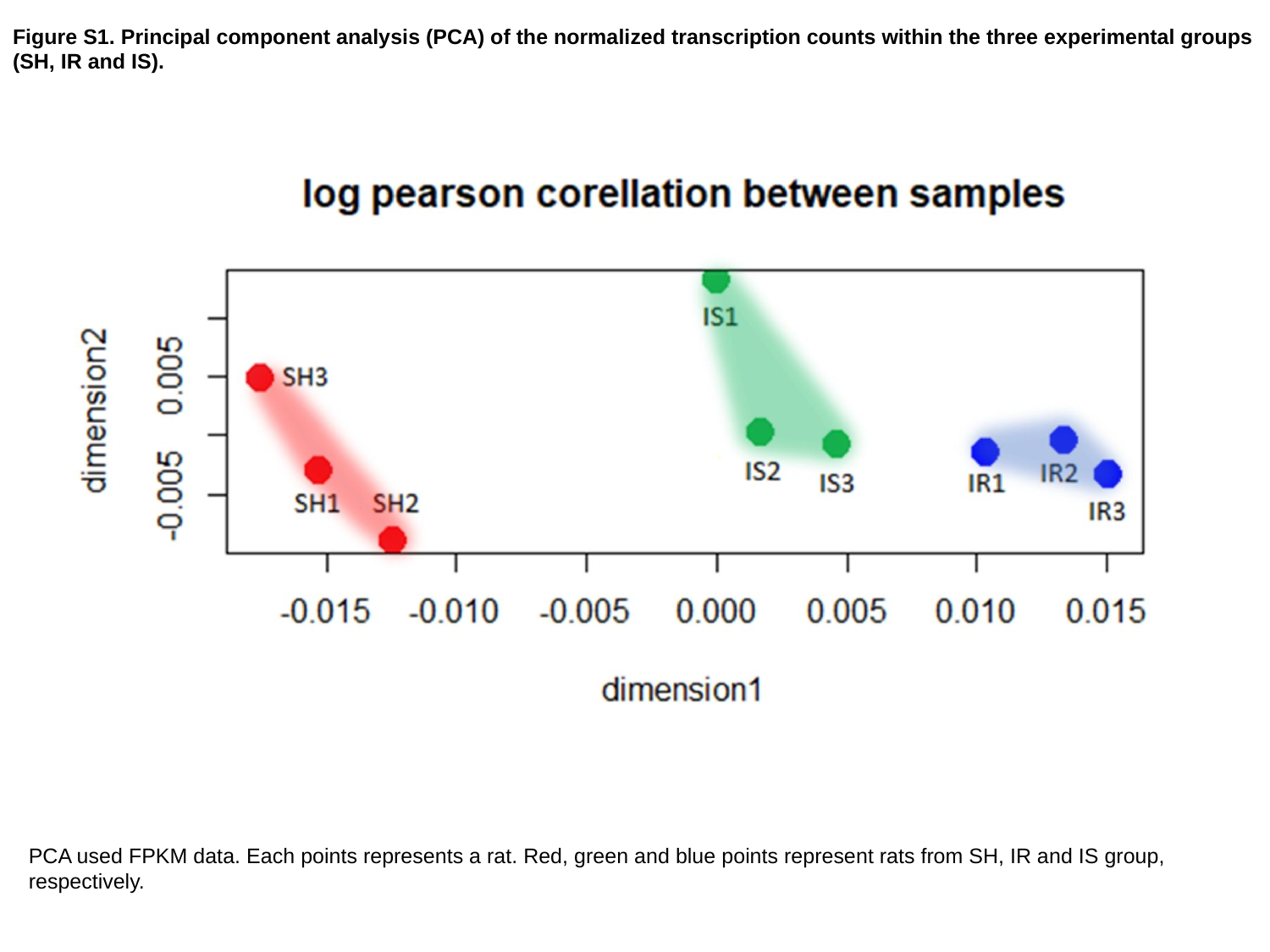

Figure S1. Principal component analysis (PCA) of the normalized transcription counts within the three experimental groups (SH, IR and IS).
PCA used FPKM data. Each points represents a rat. Red, green and blue points represent rats from SH, IR and IS group, respectively.
