# Supplementary material for: Insight into Glyproline Peptides’ Activity through the Modulation of the Inflammatory and Neurosignaling Genetic Response Following Cerebral Ischemia–Reperfusion
Source: Genes (Basel). 2022 Dec 16;13(12):2380. doi: 10.3390/genes13122380 (PMC9777888; doi:10.3390/genes13122380)
Supplement: Supplementary file 1 [file genes-13-02380-s001.zip › Supplementary Figure S2.pptx]

## Slide 1
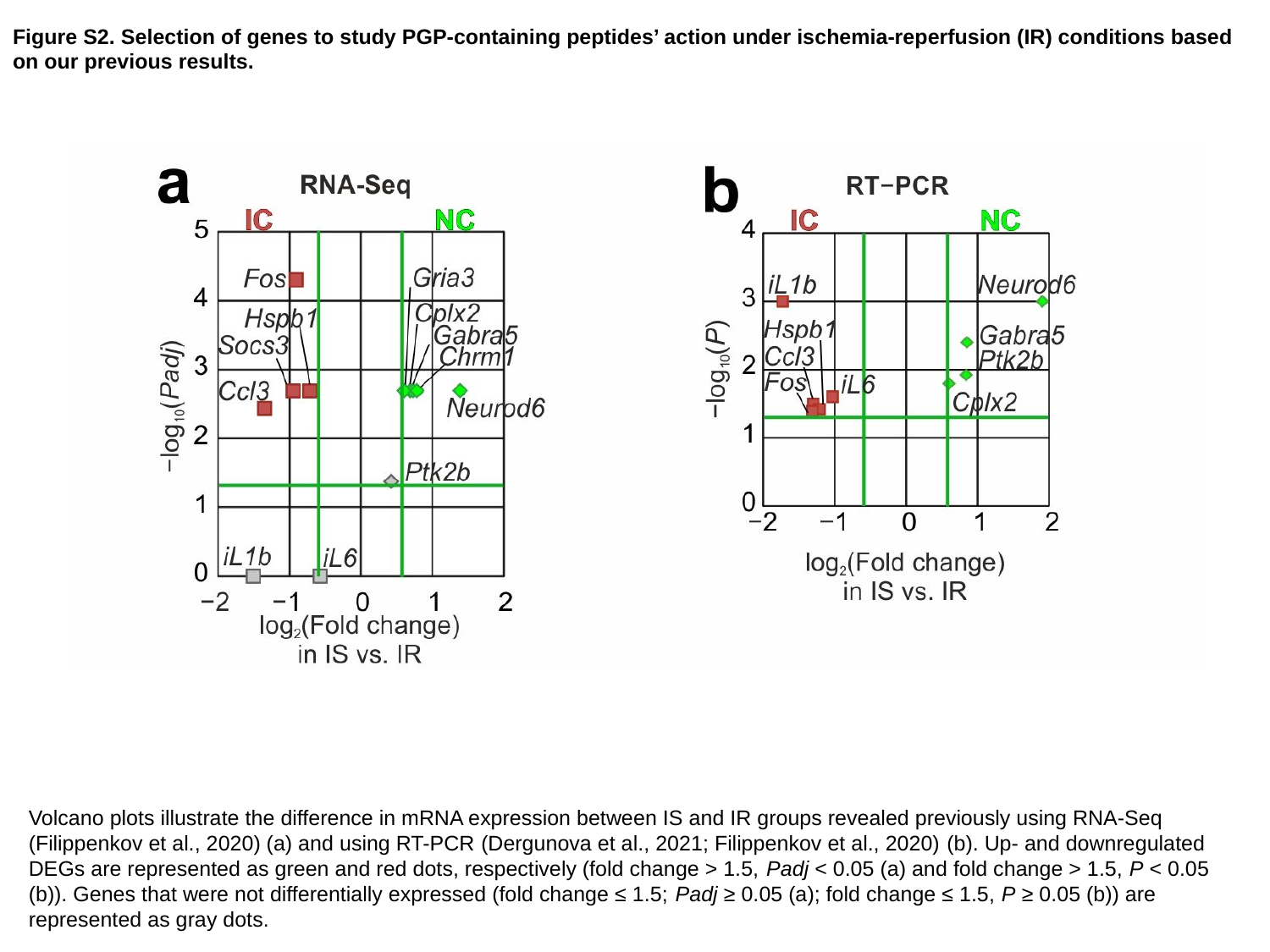

Figure S2. Selection of genes to study PGP-containing peptides’ action under ischemia-reperfusion (IR) conditions based on our previous results.
Volcano plots illustrate the difference in mRNA expression between IS and IR groups revealed previously using RNA-Seq (Filippenkov et al., 2020) (a) and using RT-PCR (Dergunova et al., 2021; Filippenkov et al., 2020) (b). Up- and downregulated DEGs are represented as green and red dots, respectively (fold change > 1.5, Padj < 0.05 (a) and fold change > 1.5, P < 0.05 (b)). Genes that were not differentially expressed (fold change ≤ 1.5; Padj ≥ 0.05 (a); fold change ≤ 1.5, P ≥ 0.05 (b)) are represented as gray dots.
